# Dynamic prognostication using conditional survival analysis for patients with operable lung adenocarcinoma

**DOI:** 10.18632/oncotarget.12920

**Published:** 2016-10-26

**Authors:** Wooil Kim, Ho Yun Lee, Sin-Ho Jung, Min-Ah Woo, Hong Kwan Kim, Yong Soo Choi, Jhingook Kim, Jae Ill Zo, Young Mog Shim, Joungho Han, Ji Yun Jeong, Joon Young Choi, Kyung Soo Lee

**Affiliations:** ^1^ Department of Radiology and Center for Imaging Science, Samsung Medical Center, Sungkyunkwan University School of Medicine, Seoul, Korea; ^2^ Biostatistics and Clinical Epidemiology Center, Samsung Medical Center, Sungkyunkwan University School of Medicine, Seoul, Korea; ^3^ Department of Thoracic Surgery and Cardiovascular Surgery, Samsung Medical Center, Sungkyunkwan University School of Medicine, Seoul, Korea; ^4^ Department of Pathology, Samsung Medical Center, Sungkyunkwan University School of Medicine, Seoul, Korea; ^5^ Department of Pathology, Kyungpook National University Medical, Center, Kyungpook National University School of Medicine, Daegu, Korea; ^6^ Department of Nuclear Medicine, Samsung Medical Center, Sungkyunkwan University School of Medicine, Seoul, Korea

**Keywords:** conditional survival, lung adenocarcinoma, TDR, SUVmax

## Abstract

**Purpose:**

To evaluate conditional survival among patients with surgically resected stage I-IIIa lung adenocarcinoma and identify changes in prognostic contributions for various prognostic factors over time.

**Patients and Methods:**

We performed conditional survival analysis at each t_0_ (=0, 1, 2, 3, 4, 5 years) for 723 consecutive patients who underwent surgical resection for lung adenocarcinoma, stratified by various clinico-demographic features, as well as pathologic and imaging (tumor-shadow disappearance ratio [TDR] on CT and maximum standardized uptake value [SUVmax] on PET) characteristics. Uni- and multivariableCox regression analyses were performed to evaluate relationships between those variables and conditional survival.

**Results:**

Three-year conditional overall survival (OS) and disease-free survival (DFS) were 92.12% and 75.51% at baseline, but improved steadily up to 98.33% and 95.95% at 5 years after surgery. In contrast to demographic factors, pathologic (stage, subtype, pathologic grade and differentiation) and radiologic factors (TDR and SUVmax) maintained a statistically significant association with subseqeunt 3-year OS until 3 years after surgery. According to the multivariableanalysis, high SUVmax and low TDR value were independent predictors of subsequent 3-year OS and DFS at baseline, 1 and 2 years after surgery, respectively.

**Conclusion:**

Our findings based on CS provide theoretical background for clinicians to plan longer period of surveillance following lung adenocarcinoma resection in survivors with preoperatively high SUVmax and low TDR on PET-CT and chest CT, respectively.

## INTRODUCTION

Lung cancer patients need accurate and integrated information about risk of recurrence and survival to help with informed decision-making. Traditional survival estimates are given by survival from the time of diagnosis in most reports, representing cumulative survival. However, probabilities of disease recurrence and death evolve over time and usually decline with increased survivorship. As a result, cumulative survival estimates calculated at the time of initial diagnosis have limited utility for follow-up care, since they provide only a static view of risk without postoperative follow-up information and do not reflect changes in prognosis over time.

Conditional survival (CS), derived from the concept of conditional probability, is an estimate of survival probability after having already survived for a specific time after a cancer diagnosis [1]. This estimate of survival is clinically relevant because it reflects the change in survival likelihood with increasing duration of follow-up from the time of the initial cancer diagnosis. CS analysis has been reported for various kinds of malignancies, including ovarian cancer, colon cancer and GIST [2–4]. Also, some studies comprehensively reported CS of non-small cell lung cancer (NSCLC) without specific focus on lung adenocarcinoma which is the most common histologic type of NSCLC [5–8].

In this study, we assessed conditional overall survival (OS) and disease-free survival (DFS) among patients with surgically resected stage I-IIIa lung adenocarcinoma and compared the results with traditional survival estimates. Moreover, recently highlighted prognostic factors of lung adenocarcinoma from the International Association for the Study of Lung Cancer/American Thoracic Society/European Respiratory Society (IASLC/ATS/ERS) lung adenocarcinoma classification scheme [9, 10] and image biomarkers such as tumor-shadow disappearance ratio (TDR) on CT and maximum standardized uptake value (SUVmax) on 18F-fluoro-2-deoxyglucose (FDG)-PET/CT [11] were evaluated together with traditionally well-known clinico-pathologic factors to identify changes in prognostic contribution over time for each factor.

## PATIENTS AND METHODS

Our institutional review board approved this retrospective study and informed consent was waived (No. 2015-11-009).

### Study population and data collection

Eight-hundred and thirty consecutive patients with lung adenocarcinoma were identified between September 2003 and August 2011. All patients underwent complete resection and mediastinal lymph node dissection at Samsung Medical Center (Seoul, Korea) with or without postsurgical adjuvant therapy. Both chest CT and integrated FDG PET/CT were obtained within the month before resection from all patients.

Among these, 68 patients were excluded by prognosis-related factors such as presence of another cancer (43 patients) and micrometastasis at the time of surgery (25 patients). Another 39 patients were excluded due to insufficient pathologic slides for evaluation of the whole tumor. Ultimately, 723 patients (372 males, 351 females; median age, 61 years) were included in this study (Figure A1).

**Figure 1:**
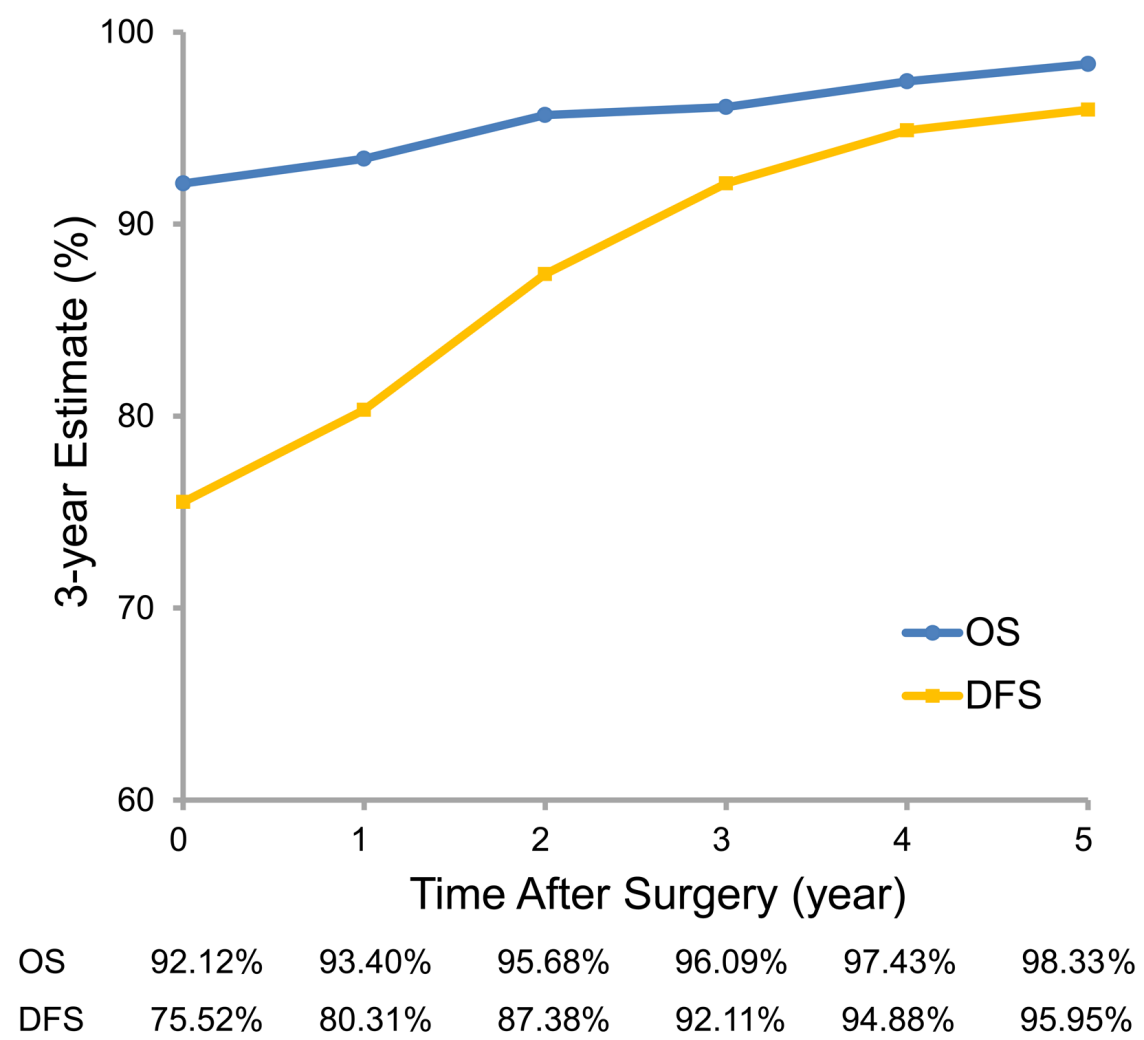
Three-year conditional overall and disease-free survival estimates

All cases were staged according to the seventh edition of the TNM classification for lung cancer [12, 13]. Pathologic subtyping was carried out according to the criteria of the IASLC/ATS/ERS classification scheme in all cases [9]. For pathologic grading, we adopted a recently proposed system [10] categorizing adenocarcinoma-in-situ, minimally invasive adenocarcinoma and lepidic adenocarcinoma as low grade, acinar and papillary tumor as intermediate grade, and solid and micropapillary tumors as high grade. In addition, according to World Health Organization classification criteria [14], histologic differentiation was categorized into poorly, moderately or well differentiated carcinomas.

### Imaging analysis

PET/CT imaging was evaluated by a nuclear medicine physician who was unaware of clinical and pathologic data. FDG uptake was evaluated by placing regions of interest and calculated as the SUVmax.

Two chest radiologists were asked to retrospectively evaluate CT scans for maximal diameter of nodules and TDR without clinical information, PET findings, and histologic diagnoses. The longest tumor diameter was measured manually on lung window images of PACS monitors using electronic measurement tools on transverse images. For calculation of TDR [15], the maximum dimension of the tumors and the largest dimension perpendicular to the maximum diameter were measured using both the lung and mediastinal windows. In addition, the observers assessed tumor solidity to visually classify density of the lesions into three categories (non-solid, part-solid, and solid feature) using both lung and mediastinal window settings.

### Statistical analysis

To calculate conditional OS and DFS after surgical resection of lung adenocarcinoma, electronic medical records were reviewed for date of last follow-up, documented recurrence, or death. Recurrence was defined as any documented clinical or pathologic evidence of local or distant disease recurrence. DFS was defined as the time from surgery to the first event of recurrence or the last follow up visit. In this study, we evaluated 3-year conditional survival, facilitating prognostication for survivors who have already passed through high risk period of recurrence [16–18].

Conditional survival probability of time t conditioning at t_0_ is the probability that patients who are alive at time t_0_ survive for additional t. Conditional survival analysis at time t_0_ (≥0) was conducted by applying standard survival analysis methods such as the Kaplan-Meier method, the log-rank test and Cox regression method, to data sets consisting of subjects at risk at time t_0_. Three-year conditional OS and DFS estimates were computed within subgroups defined by performance status, sex, smoking history, stage, pathologic factors, radiologic factors, and history of adjuvant therapy. We also evaluated the contribution of patient characteristics on OS and DFS at baseline and on conditional OS and DFS at 1, 2, 3, 4 and 5 years after surgery using univariable Cox regression models to calculate hazard ratios and corresponding 95% CIs. The size of some of the subgroups and the number of events was too small to yield meaningful results for later years. Furthermore, to determine independent prognostic predictors and to quantify temporal changes of contribution of such predictors on patient prognosis over time, multivariable conditional Cox regression models were fitted at each t_0_ ( = 0, 1, 2, 3, 4, 5 years) using predictors selected with a stepwise method. For a chosen time point t0, we considered the set of all predictor which had been selected for at least one t0 value in order to investigate the time effect of a predictor. Also, the hazard ratio estimates were plotted over the range of t_0_ values. For each significant continuous predictor, a cutoff value was chosen by fitting a conditional univariable Cox regression model for each t_0_ value and finding a value that optimally split all patients into two groups in terms of p-value and hazard ratio in common over different t_0_ values. Conditional 3-year OS and DFS were estimated for each patient group defined by each binary predictor and plotted over different t_0_ values. These analyses were conducted using SAS version 9.4 (SAS Institute, Cary, NC) and R 2.10.0 (Vienna, Austria; http://www.R-project.org).

## RESULTS

### Baseline demographics and conditional survival analysis

Patient characteristics and radiological features are summarized in the Supplementary Table. After a median follow-up of 3.8 years (range 0.04-9.58 years), 57 (7.8%) patients died (41 and 16 subjects with and without recurrences or metastases, respectively) and 177 (24.48%) recurrences or metastases were recorded after surgical resection. Among all 723 patients included in this study, median OS and DFS were 3.59 years (range, 0.04-9.58 years) and 2.93 years (range, 0.04-9.58), respectively.

The 3-year OS and DFS were 92.12% and 75.52% at baseline, respectively. The probability of surviving an additional 3 years, conditioned on having already survived 1, 2, 3, 4, and 5 years after surgery, improved to 93.40%, 95.68%, 96.09%, 97.43% and 98.33%, respectively. The probability of surviving an additional 3 years without recurrence, conditioned on having already survived 1, 2, 3, 4, and 5 years after surgery, was 80.31%, 87.38%, 92.11%, 94.88% and 95.95%, respectively. Figure 1 summarizes the conditional probabilities of 3-year OS and DFS for each time point.

### Distribution of clinico-pathologic characteristics and stratified conditional survival analysis across years

Table 1 shows the distribution of disease and clinical characteristics among patients across years of OS and DFS. In general, the ratio of male sex, ever-smoker, advanced disease stage, high pathologic grade and poor differentiation diminished over time, suggesting that those features correlated to poor OS and DFS. Conversely, proportion of female sex, never-smoker, early disease stage, low pathologic grade and well differentiation gradual increased over time.

**Table 1:** Distribution of clinico-pathologic characteristics across years of overall (OS) and disease-free survival (DFS)

	Baseline		1-year				2-year				3-year				4-year				5-year			
	*N*=723		OS (*N*=682)		DFS (*N*=640)		OS (*N*=602)		DFS (*N*=523)		OS (*N*=435)		DFS (*N*=355)		OS (*N*=311)		DFS (*N*=254)		OS (*N*=180)		DFS (*N*=148)	
Characteristic	No.	%	No.	%	No.	%	No.	%	No.	%	No.	%	No.	%	No.	%	No.	%	No.	%	No.	%
**Performance Status**																						
0	318	44	303	44.4	297	46.4	274	45.5	253	48.4	211	48.5	185	52.1	163	52.4	140	55.1	105	58.3	87	58.8
1	386	53.4	363	53.2	327	51.1	316	52.5	260	49.7	218	50.1	166	46.8	145	46.6	111	43.7	74	41.1	60	40.5
2	16	2.21	14	2.05	14	2.19	10	1.66	8	1.53	5	1.15	4	1.13	3	0.96	3	1.18	1	0.56	1	0.68
3	3	0.41	2	0.29	2	0.31	2	0.33	2	0.38	1	0.23	0	0	0	0	0	0	0	0	0	0
**Age**																						
Median	60		59.9		60		60		60		59.7		60		60.3		61.0		59.0		59.2	
Interquartile range	13.6		13		13		13		13		13.2		13.0		12.3		12.5		11.9		11.8	
**Sex**																						
Male	372	51.5	343	50.3	312	48.8	294	48.8	255	48.8	203	46.7	165	46.5	133	42.8	109	42.9	73	40.6	60	40.5
Female	351	48.6	339	49.7	328	51.3	308	51.2	268	51.2	232	53.3	190	53.5	178	57.2	145	57.1	107	59.4	88	59.5
**Smoking history**																						
Never-smoker	420	58.1	403	59.1	386	60.3	371	61.6	322	61.6	281	64.6	228	64.2	213	68.5	175	68.9	132	73.3	109	73.7
Ever-smoker	303	41.9	279	40.9	254	39.7	231	38.4	201	38.4	154	35.4	127	35.8	98	31.5	79	31.1	48	26.7	39	26.4
**T category**																						
T1a	333	46.1	318	46.6	309	48.3	283	47	267	51.1	218	50.1	194	54.7	166	53.4	148	58.3	95	52.8	85	57.4
T1b	209	28.9	202	29.6	190	29.7	184	30.6	154	29.5	133	30.6	106	29.9	97	31.2	74	29.1	63	35	45	30.4
T2a	140	19.4	128	18.8	116	18.1	110	18.3	86	16.4	72	16.6	50	14.1	41	13.2	28	11	19	10.6	17	11.5
T2b	31	4.29	26	3.81	20	3.13	18	2.99	12	2.29	11	2.53	4	1.13	6	1.93	3	1.18	3	1.67	1	0.68
T3a	10	1.38	8	1.17	5	0.78	7	1.16	4	0.76	1	0.23	1	0.28	1	0.32	1	0.39	0	0	0	0
**N category**																						
N0	581	80.4	557	81.7	535	83.6	498	82.7	452	86.4	360	82.8	314	88.5	268	86.2	229	90.2	165	91.7	139	93.9
N1	82	11.3	76	11.1	67	10.5	62	10.3	47	8.99	49	11.3	27	7.61	31	9.97	20	7.87	13	7.22	8	5.41
N2	60	8.3	49	7.18	38	5.94	42	6.98	24	4.59	26	5.98	14	3.94	12	3.86	5	1.97	2	1.11	1	0.68
**Stage**																						
IA	471	65.2	453	66.4	440	68.8	405	67.3	375	71.7	301	69.2	270	76.1	232	74.6	203	79.9	146	81.1	122	82.4
IB	88	12.2	84	12.3	78	12.2	76	12.6	65	12.4	50	11.5	39	11	30	9.65	22	8.66	17	9.44	16	10.8
IIA	90	12.5	84	12.3	77	12	71	11.8	55	10.5	55	12.6	31	8.73	35	11.3	23	9.06	14	7.78	9	6.08
IIB	10	1.38	10	1.47	6	0.94	7	1.16	4	0.76	3	0.69	1	0.28	2	0.64	1	0.39	1	0.56	0	0
IIIA	64	8.85	51	7.48	39	6.09	43	7.14	24	4.59	26	5.98	14	3.94	12	3.86	5	1.97	2	1.11	1	0.68
**Pathologic Subtype**																						
AIS	35	4.84	35	5.13	35	5.47	35	5.81	35	6.69	31	7.13	31	8.73	25	8.04	25	9.84	11	6.11	11	7.43
MIA	34	4.7	33	4.84	33	5.16	31	5.15	31	5.93	21	4.83	21	5.92	7	2.25	7	2.76	5	2.78	5	3.38
Lepidic	125	17.3	118	17.3	116	18.1	107	17.8	106	20.3	78	17.9	75	21.1	68	21.9	63	24.8	45	25	42	28.4
Acinar	314	43.4	296	43.4	279	43.6	264	43.9	221	42.3	205	47.1	156	43.9	149	47.9	113	44.5	89	49.4	70	47.3
Papillary	65	8.99	62	9.09	53	8.28	53	8.8	40	7.65	34	7.82	21	5.92	17	5.47	12	4.72	5	2.78	3	2.03
Micropapillary	23	3.18	23	3.37	22	3.44	19	3.16	13	2.49	12	2.76	7	1.97	3	0.96	1	0.39	3	1.67	1	0.68
Solid	113	15.6	101	14.8	88	13.8	79	13.1	63	12.1	40	9.2	31	8.73	28	9	21	8.27	8	4.44	7	4.73
Variant	14	1.94	14	2.05	14	2.19	14	2.33	14	2.68	14	3.22	13	3.66	14	4.5	12	4.72	14	7.78	9	6.08
**Pathologic grade**																						
Low	194	27.4	186	27.8	184	29.4	173	29.4	172	33.8	130	30.9	127	37.1	100	33.7	95	39.3	61	36.8	58	41.7
Intermediate	379	53.5	358	53.6	332	53	317	53.9	261	51.3	239	56.8	177	51.8	166	55.9	125	51.7	94	56.6	73	52.5
High	136	19.2	124	18.6	110	17.6	98	16.7	76	14.9	52	12.4	38	11.1	31	10.4	22	9.09	11	6.63	8	5.76
**Differentiation**																						
WD	248	34.3	240	35.2	237	37	219	36.4	214	40.9	175	40.2	168	47.3	139	44.7	130	51.2	83	46.1	74	50
MD	340	47	321	47.1	299	46.7	291	48.3	236	45.1	206	47.4	147	41.4	131	42.1	94	37	86	47.8	64	43.2
PD	135	18.7	121	17.7	104	16.3	92	15.3	73	14	54	12.4	40	11.3	41	13.2	30	11.8	11	6.11	10	6.76
**Solidity**																						
Non-solid	152	21	144	21.1	144	22.5	128	21.3	127	24.3	87	20	85	23.9	62	19.9	60	23.6	26	14.4	25	16.9
Part-solid	83	11.5	80	11.7	79	12.3	78	13	76	14.5	74	17	70	19.7	69	22.2	62	24.4	56	31.1	48	32.4
Solid	488	67.5	458	67.2	417	65.2	396	65.8	320	61.2	274	63	200	56.3	180	57.9	132	52	98	54.4	75	50.7
**TDR**																						
Median	33.6		34		35.4		34		39.1		35.7		47.6		37.8		50.5		40.8		52.0	
Interquartile range	70.1		69.6		72.5		69.8		74.6		67.9		73.5		68.3		71.3		56.4		57.3	
**SUV**max																						
Median	5.10		5.10		4.65		4.95		3.90		4.60		3.30		4.10		3.10		3.40		2.85	
Interquartile range	7.30		7.30		6.80		7.30		6.35		6.58		5.60		6.38		5.55		5.45		5.00	
**Adjuvant therapy**																						
Yes	271	37.5	255	37.4	219	34.2	227	37.7	160	30.6	170	39.1	100	28.2	108	34.7	60	23.6	52	28.9	30	20.3
No	452	62.5	427	62.6	421	65.8	375	62.3	363	69.4	265	60.9	255	71.8	203	65.3	194	76.4	128	71.1	118	79.7

Abbreviations: AIS, Adenocarcinoma-In-Situ; MD, Moderately-Differentiated; MIA, Minimally Invasive Adenocarcinoma; TDR, Tumor shadow-Disappearance Ratio; PD, Poorly-Differentiated; WD, Well-Differentiated

### Cox regression analyses

Univariable Cox regression analyses evaluated the contribution of various clinical, pathological and radiological characteristics on 3-year OS and DFS at baseline and on subsequent 3-year conditional OS and DFS at 1, 2, 3, 4 and 5 years (Table 2). In terms of conditional OS, performance, stage, pathologic subtype, pathologic grade, differentiation, TDR value, SUVmax and history of adjuvant treatment significantly correlated with subsequent 3-year conditional OS until 3 years after surgery. In contrast, age, sex, smoking history and pathologic pattern group lost their statistical significance at 1 or 2 years after surgery (see Table 3). In terms of conditional DFS, performance, differentiation, TDR value, SUVmax and history of adjuvant treatment showed significant correlation with subsequent 3-year conditional DFS until 2 years after surgery. Stage, pathologic subtype and pattern group maintained their statistical significance until 1 year after surgery. None of features except age was significantly associated with 3-year DFS estimates at 3 years after surgery

**Table 2:** Univariable analyses for conditional overall (OS) and disease-free survival (DFS)

	Baseline				1-year				2-year				3-year				4-year				5-year			
	OS		DFS		OS		DFS		OS		DFS		OS		DFS		OS		DFS		OS		DFS	
Number of events (Total patients)	0 (723)		0 (723)		12 (682)		51 (640)		19 (602)		60 (523)		9 (435)		38 (355)		9 (311)		15 (254)		5 (180)		7 (148)	
Characteristics	HR (95% CI)	P-value	HR (95% CI)	P-value	HR (95% CI)	P-value	HR (95% CI)	P-value	HR (95% CI)	P-value	HR (95% CI)	P-value	HR (95% CI)	P-value	HR (95% CI)	P-value	HR (95% CI)	P-value	HR (95% CI)	P-value	HR (95% CI)	P-value	HR (95% CI)	P-value
Age at diagnosis	1.05 (1.02-1.08)	0.001	1.02 (1.00-1.03)	0.054	1.05 (1.02-1.08)	0.001	1.03 (0.99-1.08)	0.120	1.03 (0.99-1.07)	0.198	1.08 (1.02-1.15)	0.013	1.03 (0.97-1.08)	0.349	1.18 (1.06-1.32)	0.002	1.00 (0.92-1.08)	0.910	1.16 (0.88-1.53)	0.301	0.98 (0.86-1.12)	0.790	1.16 (0.88-1.53)	0.300
Performance status																								
0								-		-		-		-		-		-		-		-		-
1	4.45 (2.18-9.22)	< 0.001	2.56 (1.84-3.58)	< 0.001	4.21 (1.95-9.10)	< 0.001	2.11 (1.44-3.08)	< 0.001	4.00 (1.49-10.74)	0.006	2.22 (1.32-3.72)	0.003	3.43 (1.10-10.65)	0.033	2.05 (0.96-4.38)	0.064	3.73 (0.75-18.54)	0.107	1.71 (0.57-5.10)	0.337	0.72 (0.07-7.91)	0.786	1.63 (0.33-8.09)	0.554
2	14.16 (4.33-46.31)	< 0.001	2.61 (0.94-7.25)	0.066	8.85 (1.87-42.00)	0.006	3.71 (1.32-10.90)	0.013	20.43 (3.91-106.79)	< 0.001	NA	0.983	15.69 (1.72-142.99)	0.015	NA	0.989	NA	0.9955	NA	0.994	NA		NA	
3	20.9 (2.63-166.08)	0.004	7.67 (1.86-31.66)	< 0.001	NA	0.99	11.25 (2.70-46.88)	0.001	NA	0.992	39.47 (8.82-176.61)	< 0.001	NA	0.995	NA		NA		NA	0.998	NA		NA	
Sex																								
Female								-		-		-		-		-		-		-		-		-
Male	3.35 (1.83-6.13)	< 0.001	1.38 (1.03-1.86)	0.033	3.49 (1.76-6.89)	< 0.001	1.98 (0.91-4.29)	0.084	2.29 (1.02-5.15)	0.044	1.53 (0.53-4.42)	0.431	1.84 (0.70-4.85)	0.215	1.88 (0.31-11.25)	0.490	2.29 (0.55-9.58)	0.257	NA	0.998	0.70 (0.06-7.77)	0.775	NA	0.998
Smoking history																								
Never-smoker								-		-		-		-		-		-		-		-		-
Ever-smoker	2.50 (1.47-4.25)	0.001	1.34 (0.99-1.80)	0.055	2.42 (1.34-4.38)	0.003	1.95 (0.93-4.10)	0.079	1.70 (0.78-3.68)	0.180	1.84 (0.64-5.25)	0.256	1.17 (0.43-3.18)	0.752	3.15 (0.53-18.96)	0.209	1.44 (0.34-6.02)	0.621	NA	0.998	NA	0.996	NA	0.998
Stage																								
IA										-		-		-		-		-		-		-		-
IB	4.32 (1.92-9.74)	< 0.001	4.22 (1.87-9.50)	< 0.001	5.13 (2.21-11.89)	< 0.001	3.41 (1.14-10.18)	0.030	4.46 (1.59-12.56)	0.005	2.78 (0.72-10.78)	0.139	7.34 (2.21-25.37)	0.002	NA	0.996	23.67 (2.46-227.54)	0.006	NA	1.000	7.90 (0.52-35.64))	0.144	NA	1.000
IIA	5.67 (2.65-12.13)	< 0.001	5.86 (2.75-12.49)	< 0.001	6.23 (2.78-13.94)	< 0.001	7.85 (3.18-19.39)	< 0.001	4.45 (1.57-12.63)	0.005	4.61 (1.35-15.80)	0.015	5.87 (1.55-22.19)	0.009	6.43 (1.07-38.72)	0.042	20.69 (1.82-235.17)	0.015	NA	0.998	NA	0.998	NA	0.998
IIB	15.28 (4.35-53.72)	< 0.001	16.28 (4.65-56.98)	< 0.001	19.72 5.50-70.74)	< 0.001	NA	0.990	11.13 (1.39-89.35)	0.023	NA	0.998	29.17 (3.32-256.51)	0.002	NA	0.999	NA	0.998	NA		NA	.	NA	
IIIA	14.14 (6.86-29.12)	< 0.001	14.85 (7.26-30.40)	< 0.001	8.43 (3.40-20.91)	< 0.001	7.53 (2.30-24.63)	< 0.001	6.83 (2.05-22.72)	0.002	NA	0.994	8.09 (1.52-43.17)	0.014	NA	0.998	81.56 (6.43-1034.81)	0.001	NA		NA	.	NA	
T category																								
T1a								-		-		-		-		-		-		-		-		-
T1b	92.83 (1.25-6.40)	0.010	3.01 (1.33-6.81)	0.010	3.18 (1.28-7.88)		3.55 (1.21-10.38)	0.021	3.23 (1.10-9.46)	0.032	1.87 (0.54-6.45)	0.324	1.63 (0.47-5.64)	0.439	1.96 (0.28-13.96)	0.501	2.43 (0.41-14.55)	0.331	NA	0.998	NA	0.996	NA	0.998
T2a	6.13 (2.77-13.57)	< 0.001	6.16 (2.78-13.63)	< 0.001	6.87 (2.82-16.74)	< 0.001	6.53 (2.23-19.14)	< 0.001	5.41 (1.76-16.58)	0.003	2.20 (0.52-9.22)	0.282	3.81 (1.10-13.21)	0.035	NA	0.996	6.63 (1.11-39.76)	0.038	NA	0.999	NA	0.996	NA	0.999
T2b	18.86 (7.62-46.68)	< 0.001	18.97 (7.65-46.99)	< 0.001	18.69 (6.52-53.60)	< 0.001	14.25 (3.38-60.11)	< 0.001	9.29 (1.79-48.48)	0.008	6.70 (0.78-57.93)	0.084	11.41 (2.20-59.21)	0.004	26.74 (2.42-295.27)	0.007	NA	0.996	NA	1.000	NA	1.000	NA	1.000
T3a	19.69 (5.28-73.51)	< 0.001	19.29 (5.19-71.62)	< 0.001	9.62 (1.17-78.91)		NA	0.991	20.37 (2.32-179.11)	0.007	NA	0.993	NA	0.995	NA	0.999	NA	0.999	NA		NA		NA	
N category																								
N0								-		-		-		-		-		-		-		-		-
N1	4.85 (2.60-9.05)	< 0.001	5.10 (2.74-9.49)	< 0.001	4.89 (2.52-9.51)	< 0.001	4.86 (2.08-11.39)	< 0.001	3.85 (1.57-9.43)	0.003	2.97 (0.83-10.68)	0.095	3.42 (1.07-10.90)	0.038	3.19 (0.35-28.72)	0.302	6.91 (1.21-39.53)	0.030	NA	0.998	NA	0.997	NA	0.998
N2	7.24 (3.75-13.95)	< 0.001	7.75 (4.04-14.89)	< 0.001	4.37 (1.86-10.25)	< 0.001	5.03 (1.67-15.16)	0.004	3.32 (0.95-11.60)	0.060	NA	0.993	4.13 (0.89-19.22)	0.071	NA	0.995	23.63 (3.56-156.85)	0.001	NA	0.000	NA		NA	
Pathologic subtype*	-	< 0.001	-	< 0.001		< 0.001		0.005		0.014		0.451		0.040		0.882		0.116		0.977		0.061		*0.977*
Pathologic grade																								
Low								-		-		-		-		-		-		-		-		-
Intermediate	5.77 (1.76-18.88)	0.004	8.44 (4.29-16.62)	< 0.001	4.73 (1.43-15.69)	0.011	4.97 (1.14-21.75)	0.033	5.29 (1.23-22.83)	0.025	3.24 (0.70-15.00)	0.133	3.65 (0.82-16.34)	0.090	3.08 (0.34-27.63)	0.314	3.93 (0.47-32.72)	0.205	NA	0.998	NA	0.996	NA	0.998
High	15.58 (4.65-52.16)	< 0.001	11.58 (5.66-23.72)	< 0.001	12.48 (3.63-42.88)	< 0.001	13.64 (3.00-61.96)	0.001	8.43 (1.69-42.12)	0.009	4.81 (0.80-29.03)	0.087	5.21 (0.86-31.45)	0.072	NA	0.996	4.40 (0.27-71.67)	0.298	NA	1.000	NA	1.000	NA	1.000
Differentiation																								
WD								-		-		-		-		-		-		-		-		-
MD	13.22 (3.17-55.17)	< 0.001	13.74 (3.29-57.32)	< 0.001	11.43 (2.72-48.10)	< 0.001	8.19 (1.89-35.50)	0.005	9.97 (2.34-42.42)	0.002	5.92 (1.31-26.76)	0.021	6.80 (1.54-29.90)	0.011	1.85 (0.31-11.07)	0.501	6.80 (1.54-29.90)	0.011	1.85 (0.31-11.07)	0.501	6.21 (0.75-51.60)	0.091	NA	0.998
PD	30.2 (7.10-128.49)	< 0.001	29.77 (7.01-126.47)	< 0.001	22.62 (5.18-98.79)	< 0.001	14.10 (3.03-65.53)	0.001	3.38 (0.47-24.09)	0.225	1.93 (0.18-21.41)	0.591	1.94 (0.18-21.49)	0.590	NA	0.996	1.94 (0.18-21.49)	0.590	NA	0.996	4.70 (0.29-76.97)	0.278	NA	1.000
TDR values	0.97 (0.96-0.98)	< 0.001	0.97 (0.96-0.98)	< 0.001	0.97 (0.95-0.98)	< 0.001	0.97 (0.95-0.99)	< 0.001	0.97 (0.95-0.99)	0.001	0.98 (0.96-0.99)	0.044	0.97 (0.95-0.99)	0.012	0.99 (0.97-1.02)	0.578	0.98 (0.95-1.01)	0.119	1.00 (0.94-1.06)	0.866	0.98 (0.94-1.03)	0.454	1.00 (0.94-1.06)	0.866
SUVmax	1.14 (1.09-1.18)	< 0.001	1.11 (1.09-1.13)	< 0.001	1.15 (1.10-1.19)	< 0.001	1.10 (1.07-1.13)	< 0.001	1.13 (1.07-1.19)	< 0.001	1.10 (1.06-1.14)	0.008<0.001	1.12 (1.04-1.20)	0.003	1.07 (1.01-1.14)	0.030	1.10 (0.99-1.23)	0.084	1.05 (0.94-1.18)	0.356	1.28 (1.05-1.56)	0.016	1.07 (0.89-1.28)	0.488
Adjuvant therapy																								
No								-		-		-		-		-		-		-		-		-
Yes	4.45 (2.49-7.94)	< 0.001	11.1 (7.48-16.47)	< 0.001	5.43 (2.75-10.74)	< 0.001	7.01 (2.97-16.54)	< 0.001	6.01 (2.40-15.03)	< 0.001	6.43 (2.01-20.62)	0.002	6.30 (2.04-19.43)	0.001	4.62 (0.76-27.95)	0.096	16.11 (1.97-131.87)	0.010	NA	0.998	6.03 (0.55-66.64)	0.143	NA	0.998

*Log-Rank test Abbreviations: AIS, Adenocarcinoma-In-Situ; MD, Moderately-Differentiated; MIA, Minimally Invasive Adenocarcinoma; SUVmax, Maximum Standardized Uptake Value; TDR, Tumor shadow-Disappearance Ratio; PD, Poorly-Differentiated; WD, Well-Differentiated

**Table 3:** Multivariable analyses for conditional overall (OS) and disease-free survival (DFS)

	Baseline (n=551)		1 yr (*n*=526)		2 yr (*n*=462)		3yr (*n*=343)		4yr (*n*=254)		5yr (*n*=152)	
Number of events (Total patients)	0 (723)		12 (682)		19 (602)		9 (435)		9 (311)		5 (180)	
Factors related to OS	HR (CI)	*p*-value	HR (CI)	*p*-value	HR (CI)	*p*-value	HR (CI)	*p*-value	HR (CI)	*p*-value	HR (CI)	*p*-value
Performance status	2.73 (1.73-4.31)	<0.001	2.2 (1.31-3.70)	0.003	2.65 (1.37-5.13)	0.00374	2.31 (0.96-5.53)	0.06162	1.82 (0.41-8 .11)	0.4311	0.02 (0-2.23)	0.10596
Stage	1.36 (1.08-1.70)	0.00788	1.25 (0.98-1.59)	0.07455	1.13 (0.81-1.56)	0.4732	1.20 (0.80-1.80)	0.38697	1.95 (1.02-3.73)	0.0431	12.16 (0.84-176.75)	0.0673
Adjuvant treatment	1.81 (0.83-3.92)	0.1351	2.42 (1.04-5.65)	0.041	3.47 (1.14-10.56)	0.02879	4.38 (1.08-18.36)	0.0431	7.88 (0.73-84.69)	0.0883	1.44 (0.08-27.48)	0.8084
SUVmax	1.08 (1.03-1.14)	0.00182	1.10 (1.04-1.15)	<0.00021	1.08 (1.00-1.16)	0.0494955	1.05 (0.95-1.17)	0.33576	0.94 (0.76-1.16)	0.56162	1.19 (0.86-1.64)	0.2953
Sex	1.53 (0.60-3.88)	0.37081	2.21 (0.86-5.69)	0.10152	1.74 (0.55-5.56)	0.350496	1.63 (0.43-6.22)	0.4764	1.79 (0.30-10.70)	0.52152	0.91 (0.03-25.13)	0.9544
Smoking history	1.94 (0.83-4.56)	0.1291	1.38 (0.59-3.22)	0.4531	1.33 (0.43-4.15)	0.62455	1.01 (0.24-4.14)	0.99495	1.45 (0.21-9.98)	0.70859	0	0.9961

	Baseline (*n*=551)		1 yr (*n*=490)		2 yr (*n*=393)		3yr (*n*=273)		4yr (*n*=203)		5yr (*n*=126)	
Number of events (Total patients)	0 (723)		51 (640)		60 (523)		38 (355)		15 (254)		7 (148)	
Factors related to DFS	HR (CI)	*p*-value	HR (CI)	*p*-value	HR (CI)	*p*-value	HR (CI)	*p*-value	HR (CI)	*p*-value	HR (CI)	*p*-value
Performance status	1.58 (1.22-2.03)	<0.001	1.58 (1.16-2.15)	0.004	1.72 (1.09-2.72)	0.019	1.46 (0.70-3.05)	0.311	1.23 (0.43-3.56)	0.698	1.35 (0.26-7.08)	0.721
TDR	0.99 (0.98-0.99)	<0.001	0.99 (0.98-0.99)	<0.001	0.99 (0.98-1.00)	0.046	1.00 (0.98-1.01)	0.482	1.00 (0.98-1.02)	0.723	1.03 (0.997-1.061)	0.081
Adjuvant treatment	6.02 (3.86-9.39)	<0.001	6.68 (4.01-11.12)	<0.001	9.15 (4.53-18.48)	<0.001	10.54 (4.03-27.59)	<0.001	8.15 (2.04-32.66)	0.003	10.95 (1.46-82.43)	0.020
Stage	1.12 (1.00-1.25)	0.045	1.03 (0.90-1.18)	0.672	0.94 (0.77-1.16)	0.570	0.80 (0.54-1.18)	0.259	1.23 (0.70-2.16)	0.473	2.51 (1.01-6.26)	0.048

Abbreviations: SUVmax, Maximum Standardized Uptake Value; TDR, Tumor shadow-Disappearance Ratio

Statistically significant predictors of 3-year OS and DFS at baseline and subsequent 3-year conditional OS and DFS at 1, 2, 3, 4 and 5 years after surgery were evaluated, fitting multivariable Cox regression models with stepwise regression at each time point (Table 3). Temporally changing hazard ratios for OS were based on multivariable regression analysis (Figure A2, error bars depict 95% CIs). At baseline, factors significantly associated with poor overall survival were poor performance (HR = 2.73, *p* < 0.001), higher disease stage (HR = 1.36, *p*-value = 0.0078) and SUVmax (HR = 1.08, *p*-value = 0.0018). Among these variables, performance status and SUVmax remained statistically predictive of subsequent OS at 1 and 2 years after surgery (performance; HR = 2.20, *p*-value = 0.003 at 1-year; HR = 2.65, *p*-value = 0.0037 at 2-year; SUVmax; HR = 1.10, *p*-value = 0.0002 at 1-year; HR = 1.08, *p*-value = 0.0495 at 2-year). At the 3-year time point, there was no statistically significant predictor of subsequent 3-year OS. History of adjuvant treatment, sex and smoking history were not significant predictors of conditional OS at any time.

**Figure 2:**
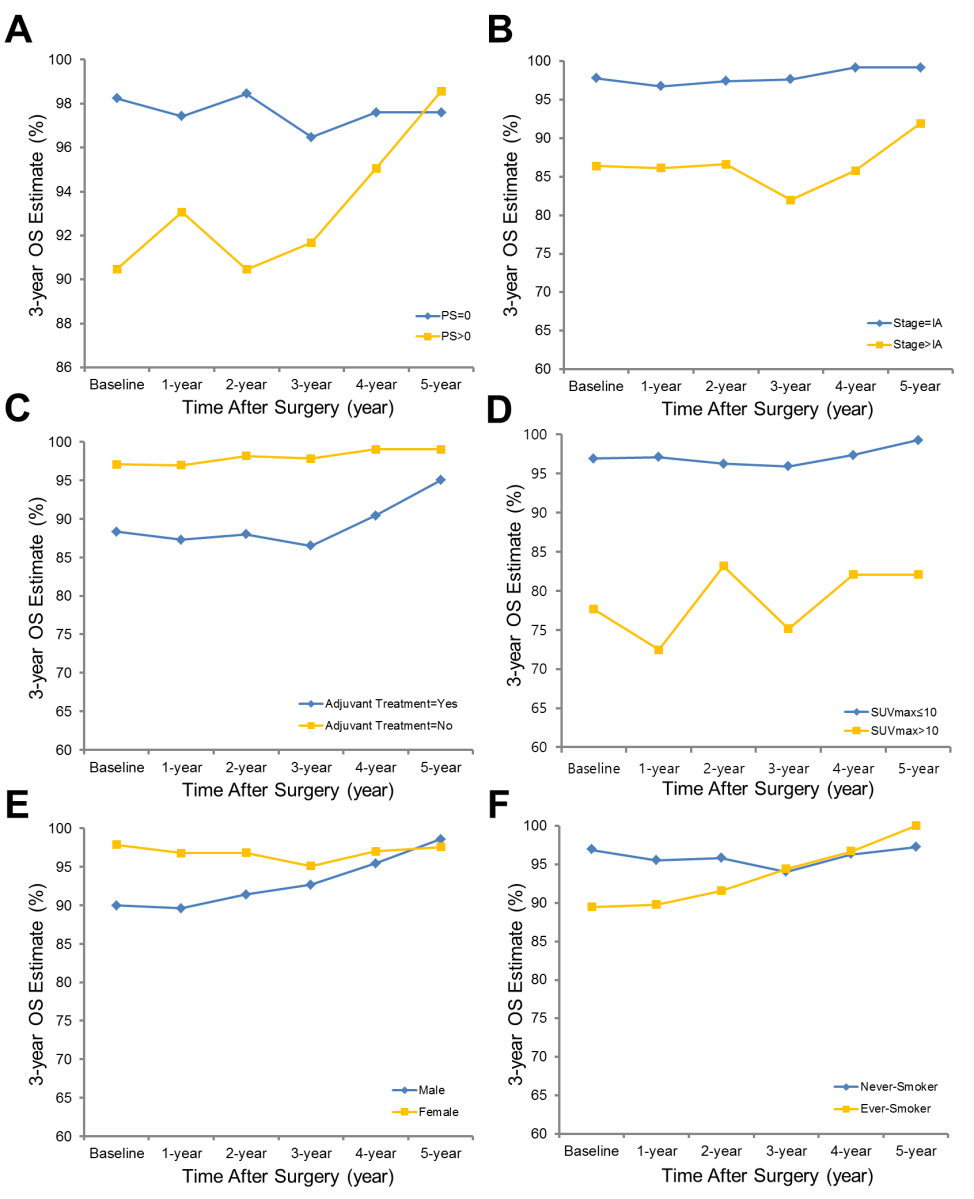
Three-year conditional overall survival estimates plotted with cut-point determination are shown, stratified by performance status **a**., stage **b**., history of adjuvant treatment **c**., SUVmax **d**., sex **e**. and history of smoking **f**. The cut-off value for SUVmax was determined as 10.

Temporally changing hazard ratios for DFS were based on multivariable regression analysis (Figure A3, error bars depict 95% CIs). At baseline, characteristics significantly associated with poor DFS included poor performance (HR = 1.58, *p*-value = 0.0004), low TDR value (HR = 0.99, *p*-value = 0.0001), history of adjuvant treatment (HR = 6.02, *p*-value < 0.0001) and higher disease stage (HR = 1.12, *p*-value = 0.0451). Among these variables, only history of adjuvant treatment remained predictive of subsequent DFS at 1, 2 and 3 years after surgery (HR = 6.68, 9.15 and 10.54, respectively). Patient performance and TDR value remained significant predictors of subsequent 3-year DFS at the 1- and 2-year mark, but lost their statistical significance by 3 years after surgery (performance; HR = 1.58, *p*-value = 0.0035 at 1 year; HR = 1.72, *p*-value = 0.0190 at 2 years; TDR value; HR = 0.99, *p*-value = 0.0006 at 1 year; HR = 0.99, *p*-value = 0.0457 at 2 years). Disease stage was no longer a significant predictor of subsequent 3-year DFS at any time point except for baseline.

**Figure 3:**
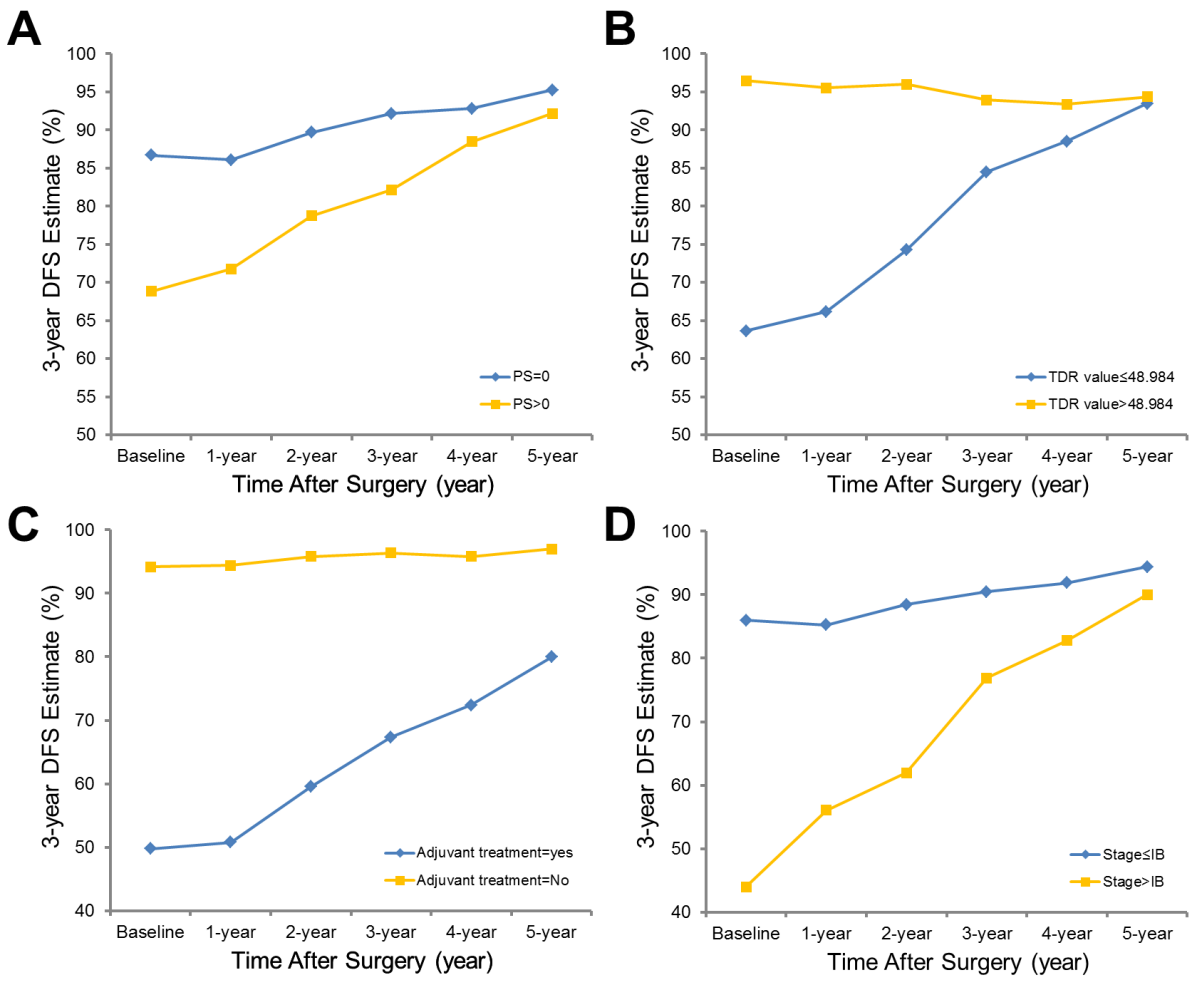
Three-year conditional disease-free survival estimates plotted with cut-point determination are shown, stratified by performance status **a**., TDR **b**., history of adjuvant treatment **c**. and stage **d**. The cut-off value for TDR was determined as 48.98.

### Stratified conditional survival probability plotting with cut-point determination

CS analysis was stratified with optimal dichotomizing cut-off value determination according to variables selected at least once during multivariable analysis, with stepwise regression at each time point (Figures 2, 3, A4 and A5). In general, 3-year OS and DFS estimates increased for all clinical, pathological and radiological features, and the gap between estimates decreased with a longer follow-up period. For example, 3-year DFS estimates for pathologic grade ranged from 66.18% to 95.36% at baseline, but this range became tighter over time and at year 5 was 87.50% to 96.55% (see Figure A5).

## DISCUSSION

The single most important prognostic factor in lung adenocarcinoma, the most common histologic type of NSCLC, has been tumor stage [8, 19]. However, even in the early stages of disease, prognosis of lung adenocarcinoma varies widely [20], necessitating the establishment of reliable prognostic factors to more accurately predict a broad spectrum of tumor behavior.

In contrast to cumulative survival calculations from traditional survival analysis that provide only a static view of risk without postoperative follow-up information, CS is more relevant to follow-up care because it reflects the change of survival likelihood with increasing duration of follow-up from the time of the initial cancer diagnosis. Leveraging the power of the CS analysis implements more evidence-drive approaches to post-therapy surveillance, particularly focused on long-term survivors over 2-3 years, which means that value of the conditional survival analysis is obviously different from traditional survival analysis and the complementary information of two different survival analyses allows for clinician into more appropriate surveillance.

To our knowledge, this is the first study assessing conditional OS and DFS among patients with lung adenocarcinoma. A few studies were previously publised regarding CS of lung cancer but those did not reflect novel lung adenocarcinoma classifcation scheme from the IASLC/ATS/ERS [5–7, 21] or included small cell lung cancer [6, 7] in conditional survival anlysis without emphasis on lung adenocarcinoma. Also, those only presented conditional survival with descriptive way, not or partially elaborating various prognostic factors including clinical, radiological and pathological aspects [5–7, 21]. Another new feature of this study is that we investigated how the effect (or significance) of each factor changed over time, rather than the time-static effect of these, on OS and DFS, which has not been revealed with conventional survival analysis (i.e., cumulative survival). Also, we focused on revealing temporal alteration of prognostic effect of previously well-known independent imaging biomarkers (TDR and SUVmax) and inter-relationship with other prognostic factors. This kind of approach is unique from already reported literatures regarding radiologic prognostic factors.

In this study, we evaluated 3-year conditional survival, facilitating prognostication for survivors who have already passed through high risk period of recurrence. We found clear differences: 3-year OS and DFS of lung adenocarcinoma patients were 92.12% and 75.51% at baseline but improved steadily up to 98.33% and 95.95%, respectively, conditioned on having already survived 1, 2, 3, 4, and 5 years after surgery (See Figure 1). Generally, we observed that OS and DFS improved most for patients with various factors known to be correlated with poor prognosis. In addition, the initial gap in OS and DFS at time of surgery between different subgroups based on performance, sex, smoking history, stage, histology and solidity on CT diminished over time, suggesting that the prognostic significance of these factors decreases as time elapses after surgery.

There have been abundant efforts to stratify patients with lung adenocarcinoma, including new classification schemes [9] or noninvasive surrogate imaging biomarkers [15, 22–26]. However, there have been no previous reports comparing prognostication capabilities and temporal changes in the contribution on prognosis after surgery for different factors. At baseline, there were significant associations between evaluated patient characteristics and survival estimates in accordance with previous studies such as age [27], performance [28], sex [29], smoking history [30], stage [13, 19], histology [9], grade [31], TDR values [32, 33], and SUVmax [34]. However, interestingly, demographic factors lost their statistical significance over time. In contrast, performance status, pathologic factors (stage, subtype, pathologic grade and differentiation), and radiologic factors (TDR and SUVmax) maintained their statistically significant association with 3-year OS until 3 years after surgery. In terms of DFS, none of features except age was significantly associated with CS estimates at 3 years after surgery, but performance, TDR values and SUVmax were statistically associated with subsequent 3-year DFS until 2 years after surgery. From these results, time-independent variables can be differentiated from time-dependent variables, for which further adjustment should be considered for more accurate estimation of prognosis and associated management.

In addition, we performed multivariable regression analysis with various demographic, pathologic and radiologic factors included to discriminate ultimate prognostic factors. Patient performance and SUVmax were independent predictors of subsequent 3-year OS at baseline, 1 and 2 years after surgery. In terms of DFS, TDR value and history of adjuvant treatment were predictive of subsequent 3-year DFS at baseline, 1 and 2 years after surgery. The cut-off values optimally splitting all patients into two groups were determined as 10 and 48.98 for SUVmax and TDR, respectively. These findings based on CS provide theoretical background for clinicians to plan longer period of surveillance following lung adenocarcinoma resection in survivors with preoperatively high SUVmax and low TDR on PET-CT and chest CT, respectively. Actually, many physicians taper follow-up frequency after 3 to 5 years, often with little justification nor evidence based on survival data, for which our study could give the answer to those uncertainties, facilitating a more evidence-based strategy for post-treatment follow-up scheduling based on actual current risk rather than simply on custom or tradition. For instance, if we suppose the condition that subsequent survival probabilities of patients with certain risk factors who survive x years from diagnosis become similar to those of patients without risk factors at diagnosis. Consequently, if clinicians follow up patients without risk factors for y years, patients with high risk factors should be comparably followed for x + y years. Therefore, surveillance strategy for lung adenocarcinoma survivors with preoperatively high SUVmax and low TDR on PET-CT and chest CT, respectively, should be tailored with longer follow-up periods, based on our result.

With respect to tumoral radiologic phenotyping, tumor metabolic information on PET contributed more to overall outcome, whereas TDR on CT contributed to treatment success or failure. Given that metabolic information indicates the degree of tumor aggressiveness [25, 26, 35] and TDR is associated with the degree of tumor invasion [35–38], imaging features observed in adenocarcinomas may provide additional prognostic information, assuming that radiologic functional phenotypes from CT and PET reflect tumor behavior.

This study was retrospectively designed with relatively small sample size and limited number of events. However, all patients underwent relatively uniform management including diagnostic work-up, treatment strategy and histopathologic evaluation solely from a single tertiary referral center in Korea, yielding a homogenous Asian study cohort with a relatively large number of subjects. Another limitation of this study is relatively short follow-up time (median 3.8 years) to precisely describe 5-year outcomes. Validation and expansion of our results with large-scale and multiracial data would allow general application.

In conclusion, conditional OS and DFS for patients with operable lung adenocarcinoma improved steadily over time. The initial gap between OS and DFS at time of surgery between different subgroups based on demographic prognostic factors diminished over time, suggesting that the prognostic significance of these factors decreases as time elapses after surgery, whereas the absolute contribution of pathologic and radiologic factors remained. Therefore, tenacious stance of clinicians on surveillance strategy after lung adenocarcinoma resection might be resonable for survivors with preoperatively high SUVmax and low TDR on PET-CT and chest CT, respectively.

## SUPPLEMENTARY MATERIALS FIGURES AND TABLES



## Abbreviations

CS: Conditional Survival
DFS: Disease-Free Survival
FDG: 18F-fluoro-2-deoxyglucose
IASLC/ATS/ERS: International Association for the Study of Lung Cancer/American Thoracic Society/European Respiratory Society
NSCLC: Non-small Cell lung Cancer
OS: Overall Survival
TDR: Tumor-shadow Disappearance Ratio
SUVmax: Maximum Standardized Uptake Value
