# Supplementary material for: Dynamic prognostication using conditional survival analysis for patients with operable lung adenocarcinoma
Source: Oncotarget. 2016 Oct 26;8(19):32201–11. doi: 10.18632/oncotarget.12920 (PMC5458278; doi:10.18632/oncotarget.12920)
Supplement: Supplementary file 1 [file oncotarget-08-32201-s001.pdf]

Dynamic prognostication using conditional survival analysis for patients with operable lung adenocarcinoma

Supplementary Material

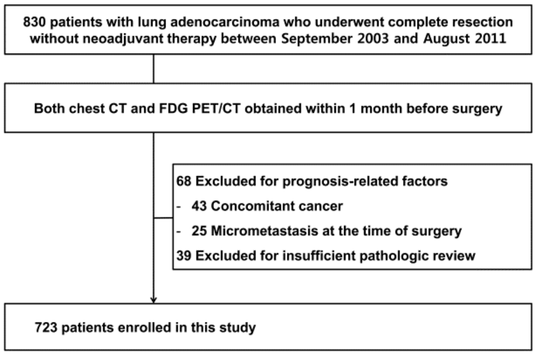

Figure - Appendix 1
Among 830 consecutive patients who underwent complete resection of lung adenocarcinoma, 68 were excluded for prognosis-related factors. Another 39 patients were excluded due to insufficient pathologic slides for evaluation of the whole tumor. Ultimately, 723 patients were included in this study.

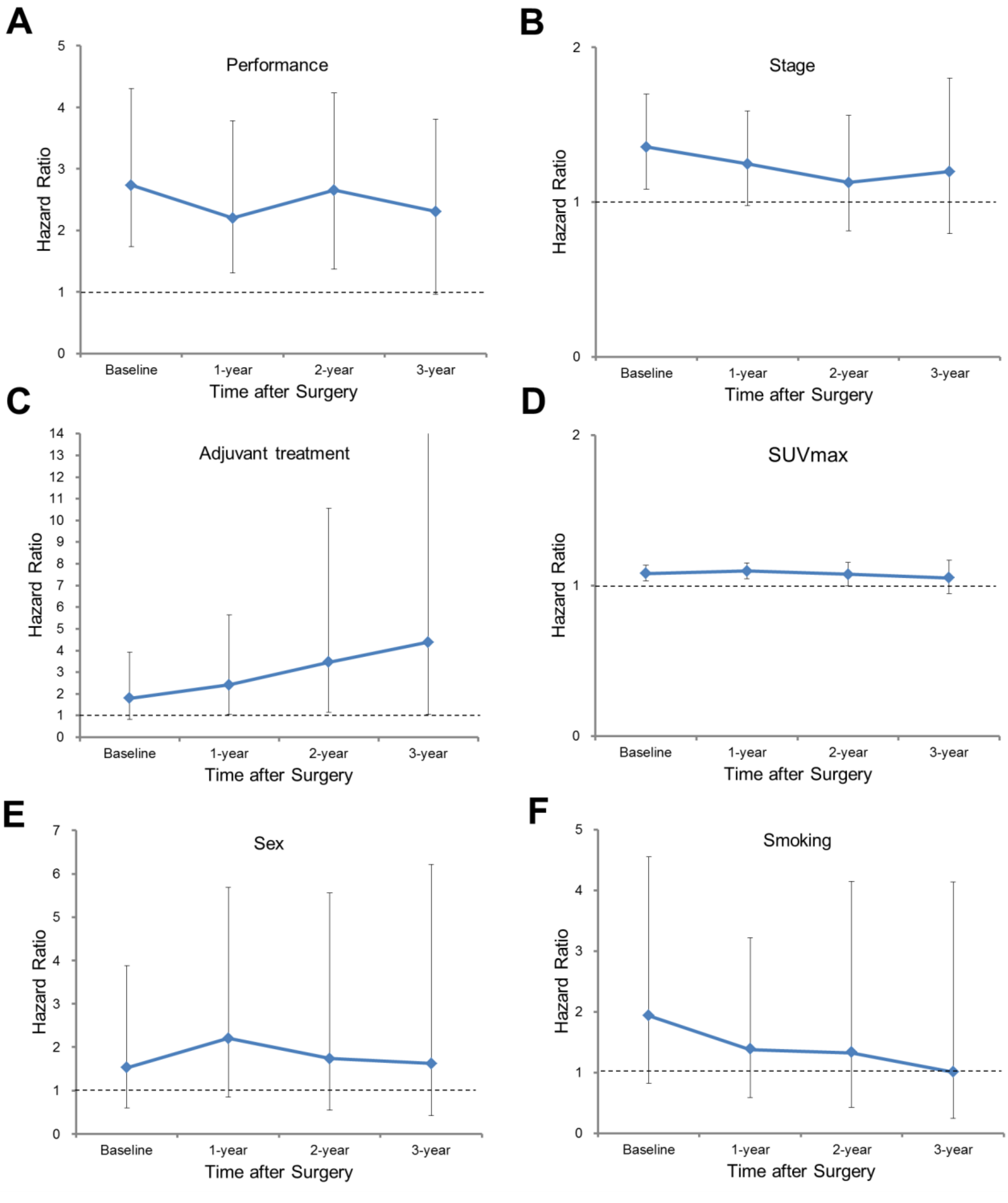

Figure - Appendix 2
Temporally changing hazard ratios of performance (a), stage (b), history of adjuvant treatment (c), SUVmax (d), sex (e) and smoking history (f) for 3-year overall survival based on multivariate regression analysis with error bars depicting 95% CIs. A dotted line is drawn at hazard ratio =1.

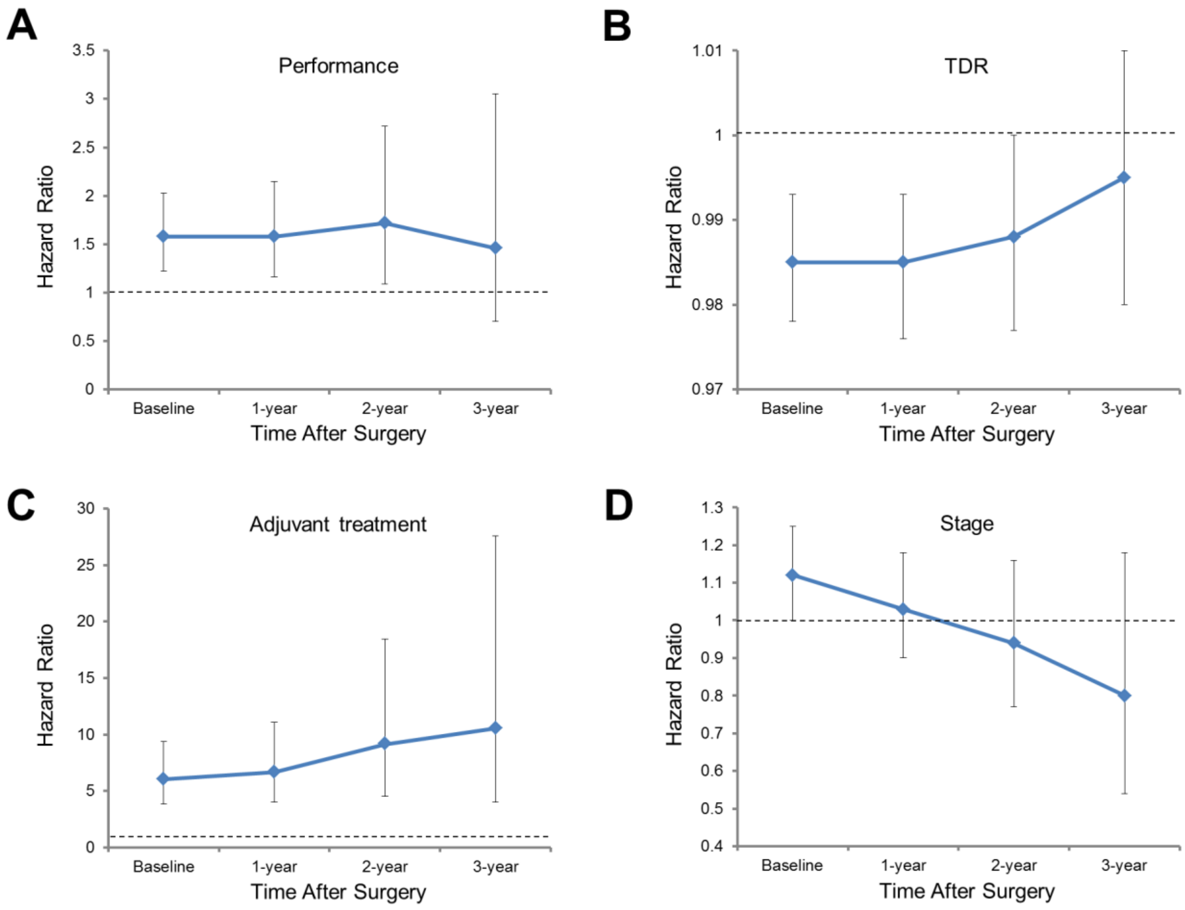

Figure - Appendix 3
Temporally changing hazard ratios of performance (a), TDR (b), history of adjuvant treatment (c) and stage (d) for 3-year disease-free survival based on multivariate regression analysis with error bars depicting 95% CIs. A dotted line is drawn at hazard ratio =1.

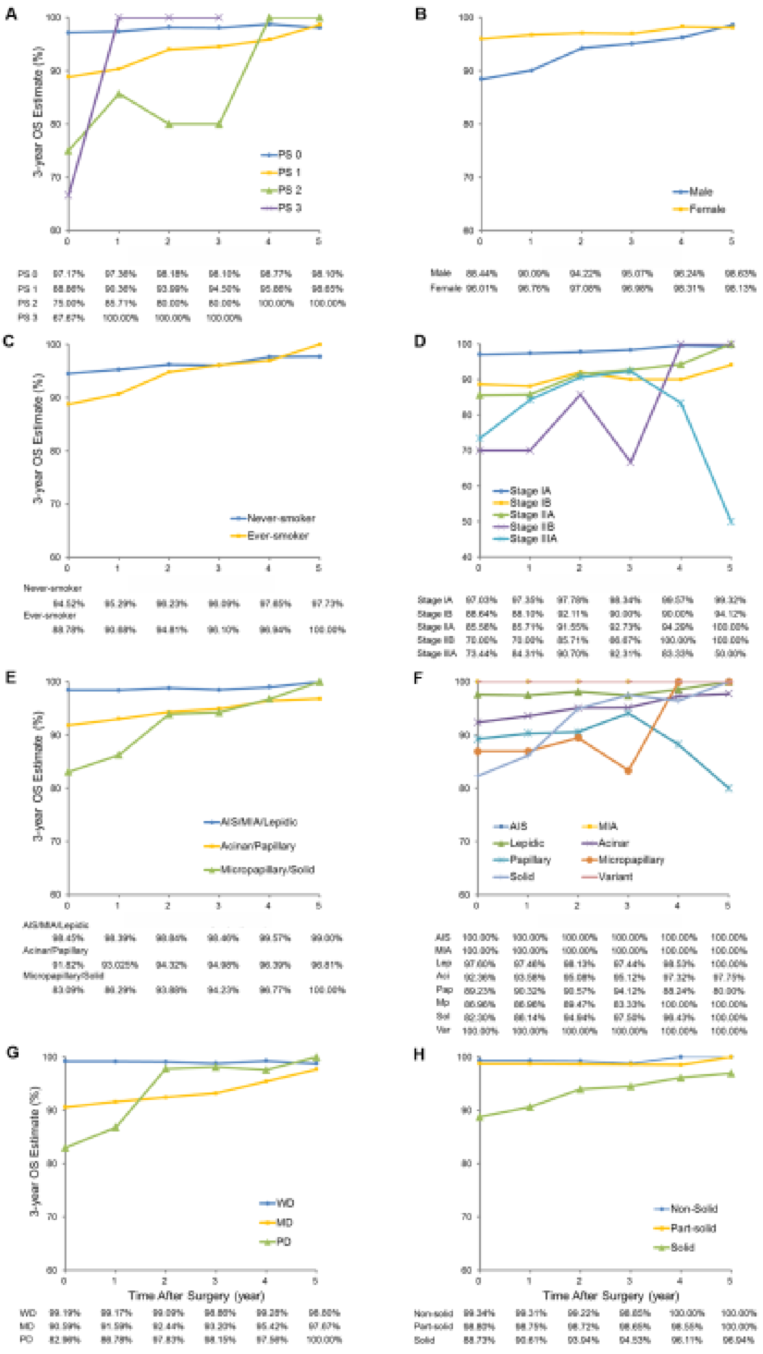

Figure - Appendix 4
Three-year conditional overall survival estimates stratified by performance status (a), sex (b), smoking history (c), stage (d), pathologic grade (e), histologic subtype (f), differentiation (g) and solidity on CT (h).

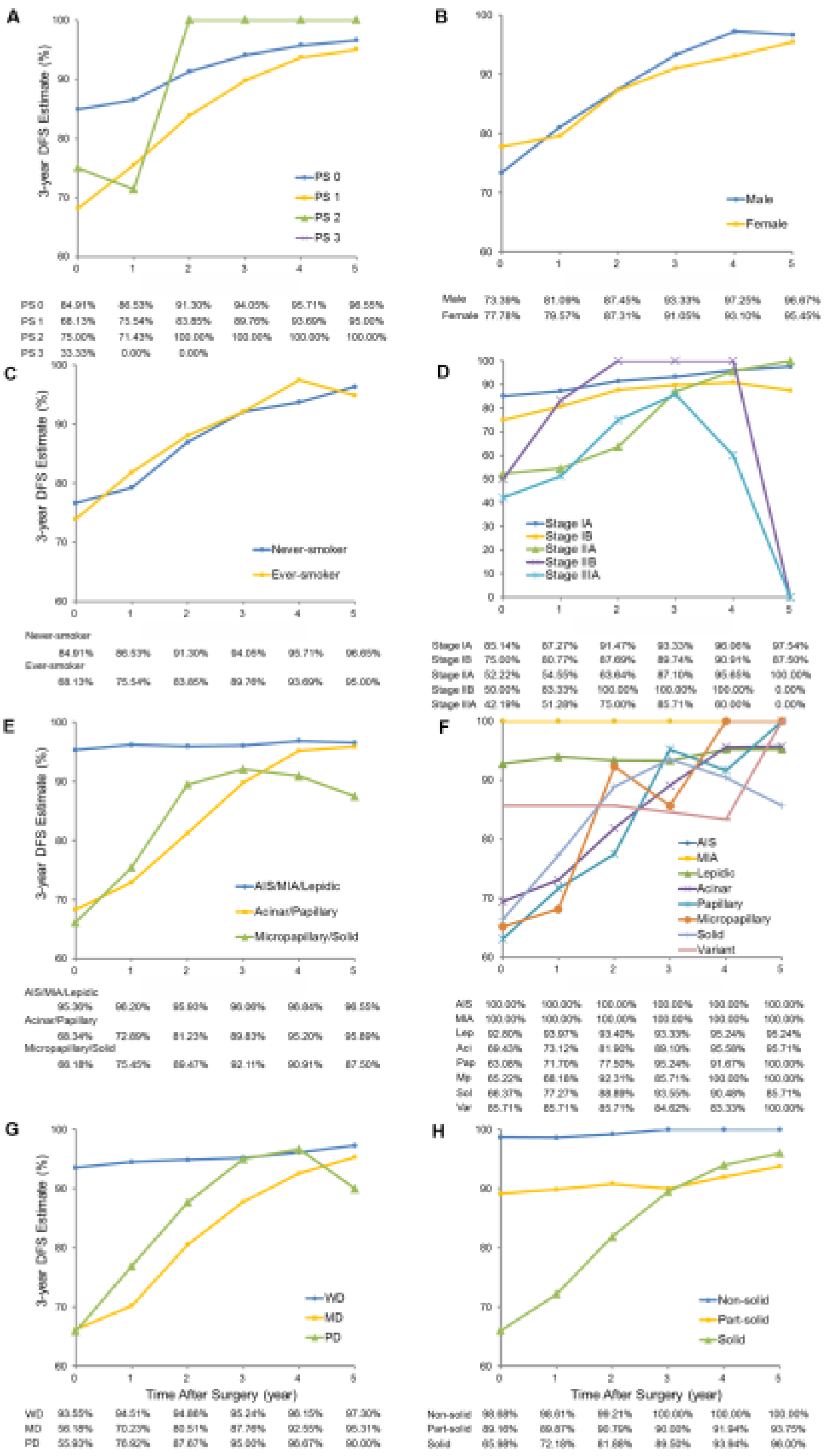

Figure - Appendix 5
Three-year conditional disease-free survival estimates stratified by performance scale (a), sex (b), smoking history (c), stage (d), pathologic grade (e), histologic subtype (f), differentiation (g) and solidity on CT (h).

Supplementary Table

Characteristics	No.	%
Age at diagnosis		
Median (value)	60	-
Range (value)	38-81	-
Sex		
Male	372	51.45
Female	351	48.55
Smoking history		
Never-smoker	420	58.09
Ever-smoker	303	41.91
Operation type		
Wedge resection	107	14.80
Lobectomy	607	83.96
Pneumonectomy	9	1.24
TDR value		
Median (value)	33.62	-
Range (value)	0.23-100	-
1st quartile	17.64	-
3rd quartile	87.82	-
Solidity		
Non-solid	152	21.02
Part-solid	83	11.48
Solid	488	67.50
SUVmax		
Median (value)	4.85	-
Range (value)	0.10-31.00	-
1st quartile	1.7	-
3rd quartile	8.95	-

Abbreviations: SUVmax, Maximum Standardized Uptake Value; TDR, tumor shadow-disappearance ratio
